# Shading Contributes to the Reduction of Stem Mechanical Strength by Decreasing Cell Wall Synthesis in Japonica Rice (*Oryza sativa* L.)

**DOI:** 10.3389/fpls.2017.00881

**Published:** 2017-05-30

**Authors:** Longmei Wu, Wujun Zhang, Yanfeng Ding, Jianwei Zhang, Elidio D. Cambula, Fei Weng, Zhenghui Liu, Chengqiang Ding, She Tang, Lin Chen, Shaohua Wang, Ganghua Li

**Affiliations:** ^1^College of Agronomy, Nanjing Agricultural UniversityNanjing, China; ^2^Key Laboratory of Crop Physiology Ecology and Production Management, Ministry of Agriculture, Nanjing Agricultural UniversityNanjing, China; ^3^Jiangsu Collaborative Innovation Center for Modern Crop Production, Nanjing Agricultural UniversityNanjing, China; ^4^Chongqing Ratooning Rice Research Center, Chongqing Academy of Agricultural SciencesChongqing, China

**Keywords:** Japonica rice (*Oryza sativa L.*), lodging, shading, sucrose, cellulose, lignin, cell wall synthesis

## Abstract

Low solar radiation caused by industrial development and solar dimming has become a limitation in crop production in China. It is widely accepted that low solar radiation influences many aspects of plant development, including slender, weak stems and susceptibility to lodging. However, the underlying mechanisms are not well understood. To clarify how low solar radiation affects stem mechanical strength formation and lodging resistance, the japonica rice cultivars Wuyunjing23 (lodging-resistant) and W3668 (lodging-susceptible) were grown under field conditions with normal light (Control) and shading (the incident light was reduced by 60%) with a black nylon net. The yield and yield components, plant morphological characteristics, the stem mechanical strength, cell wall components, culm microstructure, gene expression correlated with cellulose and lignin biosynthesis were measured. The results showed that shading significantly reduced grain yield attributed to reduction of spikelets per panicles and grain weight. The stem-breaking strength decreased significantly under shading treatment; consequently, resulting in higher lodging index in rice plant in both varieties, as revealed by decreased by culm diameter, culm wall thickness and increased plant height, gravity center height. Compared with control, cell wall components including non-structural carbohydrate, sucrose, cellulose, and lignin reduced quite higher. With histochemical straining, shading largely reduced lignin deposition in the sclerenchyma cells and vascular bundle cells compared with control, and decreased cellulose deposition in the parenchyma cells of culm tissue in both Wuyunjing23 and W3668. And under shading condition, gene expression involved in secondary cell wall synthesis, *OsPAL, OsCOMT, OsCCoAOMT, OsCCR*, and *OsCAD2*, and primary cell wall synthesis, *OsCesA1, OsCesA3*, and *OsCesA8* were decreased significantly. These results suggest that gene expression involved in the reduction of lignin and cellulose in both sclerenchyma and parenchyma cells, which attribute to lignin and cellulose in culm tissue and weak mechanical tissue, consequently, result in poor stem strength and higher lodging risks.

**Highlights**:

(1) Shading decreases the stem mechanical strength of japonica rice by decreasing non-structural carbohydrate, sucrose, lignin, and cellulose accumulation in culms.

(2) The decrease of carbon source under shading condition is the cause for the lower lignin and cellulose accumulation in culm.

(3) The expression of genes involved in lignin and primarily cell wall cellulose biosynthesis (*OsCesA1, OsCesA3*, and *OsCesA8*) at the stem formation stage are down-regulated under shading condition, inducing defective cell wall development and poor lodging resistance.

## Introduction

Rice is a photoautotrophic crop and therefore relies on sunlight to generate carbohydrates to engage in growth and development. In recent decades, with increasing aerosol emissions due to industrial pollution, solar radiation has unfortunately decreased (global dimming) with a trend of 0.54 Wm^2^yr^-1^ in South Asia (Stanhill and Cohen, 2001; Ramanathan and Feng, 2009; Kambezidis et al., 2012). Due to strong anthropogenic activities and air pollution in China, solar radiation is decreasing in most regions (Yang et al., 2013; Fu et al., 2015; Liu J. et al., 2015). This is especially true in China’s primary area, from 25°N to 45°N, where the solar radiation declined by 1.4∼2.7% per decade (Stanhill and Cohen, 2001). Nevertheless, solar dimming is reducing the light intensity within plant canopies, and the light intensity plays vital roles in stem elongation and development. Low solar radiation significantly decreases the photosynthetically active radiation (PAR) in the rice canopy. As reported, shading results in more slender stems upon elongation and reduced whole-plant mechanical stability (Huber et al., 2014); shading increased the lodging rate, and an increase in the light intensity increased the stalk strength and decreased lodging in maize (Xue et al., 2016). Therefore, we hypothesized that shading may influence rice stem mechanical strength and thus will affect susceptibility to lodging.

Lodging is defined as the permanent displacement of plant stems from a vertical position, and it is caused by a loss of balance due to many internal and natural factors. Lodging primarily occurred at the basal internode, including the third or fourth internode from the top. Many researchers have reported the relationship between lodging and related traits. Plant height reduction was successful at improving lodging resistance during the “Green Revolution” (Khush, 1999). The plant height and gravity center height were major morphological characteristics that affected lodging resistance (Chandler, 1969). However, the plant height was reportedly not the primary lodging-related factor among varieties, and the culm width and thickness were also identified as important morphological traits associated with lodging resistance in wheat (Zuber et al., 1999). In previous research, the stiffness of the basal internode and the culm width, culm thickness, and well-developed mechanical tissues contributed to stem mechanical strength or lodging resistance. Furthermore, the mechanical strength of the basal internode primarily depends on carbohydrates, such as non-structural carbohydrates and structural carbohydrates (primarily lignin and cellulose). From the start of rice grain filling, non-structural carbohydrates are generally more easily transported to grains for grain filling, so a plant’s mechanical strength primarily depends on having more structural carbohydrates (e.g., lignin and cellulose) in the basal stems (Zhang et al., 2014). As mentioned before, despite the complexity of lodging influencing factors, cellulose and lignin are two major structural carbohydrates of secondary cell walls in vascular plants, and they are well-known for being primarily responsible for stem mechanical strength. A significant correlation was found between the cellulose or lignin accumulation and the mechanical strength of rice (Zhang et al., 2014, 2016a), wheat (Peng et al., 2014), and buckwheat (Wang C. et al., 2015). The accumulation of lignin and the cellulose of culms in lodging rice varieties were lower than that in lodging-resistant varieties (Zhang et al., 2016a).

Environment factors including fertilizer, such as nitrogen application, as well as the light intensity regulate the biosynthesis and accumulation of lignin or cellulose in plants. For example, the light intensity up-regulated the phenylpropanoid (which is the origin of lignin biosynthesis) biosynthesis genes in tea callus; the phenylpropanoid accumulation increased (Wang et al., 2012b), promoting the lignin content in the cell walls of soybean hypocotyls (Su et al., 2005). It was also reported that low light intensity decreased the lignin content in tea (Wang et al., 2012a), reduced the cellulose content in cotton (Chen et al., 2014), increased lodging risk in wheat (Sparkes and King, 2008), etc. In rice, previous studies have assessed how artificial shading regulates starch pasting characteristics (Wang et al., 2013), grain filling (Wang L. et al., 2015), yield reduction (Mo et al., 2015), and internode elongation in rice (Liu et al., 2016). However, shading treatment from elongation to heading (the key period of stem formation) affects the physical strength of the rice basal internodes. The way in which lignin or cellulose biosynthesis and accumulation in culms are impacted by shading treatments is not clear. The objectives of the present study was to investigate the effects of shading on rice lodging behavior from the jointing to heading stages by observing the morphological characteristics and microstructure characteristics of the second basal internodes, non-structural carbohydrates, sucrose, lignin and cellulose biosynthesis and accumulation, and their relationships to lodging resistance.

## Materials and Methods

### Plant Materials and Growth Conditions

A field experiment was conducted at Baolin village, Danyang County, Jiangsu province (31°54 N, 119°28 E) in 2015 and 2016. The soil was an Orthic Acrisol with a total nitrogen content of 1.08, 1.20 g kg^-1^, available phosphorus of 13.5, 12.6 mg kg^-1^, available potassium of 109.8, 105.6 mg kg^-1^, organic matter of 20.8, 20.2 g kg^-1^, and a pH of 6.8, 6.6 in 2015 and 2016, respectively. The experiments were arranged in a split-plot design with three replications. The area of each plot was 24 m^2^. In each plot, the average spacing was 25 hills per m^2^ (13.3cm × 30 cm), with three seedlings per hill.

Two japonica rice cultivars, namely Wuyunjing23 (lodging-insensitive) and W3668 (lodging-sensitive), were used in this experiment. When the second basal internodes emerged (jointing stage), shading treatments were imposed on selected plots with black sun-shading net, which reduced the incident light by 60% (Shading), and there was a control with no shading (Control). The shading treatment was implemented from the elongation to the heading stages. The shading cloths were removed at the heading stage of rice population. In each year, seeds were sown in nursery boxes on 28 May and the seedlings were transplanted on 18 June in 2015 and sown in nursery boxes on 30 May and the seedlings were transplanted on 21 June in 2016. The nitrogen application rate, 180 kg N ha^-1^ as urea, was applied four times (basal fertilizer was applied 1 day before transplanting, tillering fertilizer was applied 7 days after transplanting, top-dressing nitrogen was applied at the panicle initiation stage and when the second leaf from the top was fully extended) by using 30%, 30%, 20%, and 20% in each stage, respectively. Phosphorus with 90 kg P_2_O_5_ ha^-1^ as single superphosphate was applied as basal fertilizer 1 day before transplanting, and potassium with 120 kg K_2_O ha^-1^ as potassium chloride was provided in two applications (1 day pre-transplantation and panicle initiation stage) by using 50% and 50% at each stage in all plots. Water, weeds, insects, and disease were controlled in accordance with local routine management to avoid yield loss.

### Measurement of Grain Yield and Yield Components, Culm Diameter, Culm Wall Thickness, Gravity Center Height and Plant Height

At maturity, japonica rice grain yields (14.5% moisture content) were measured within five hills according to the average tillers from each plot. The grain moisture content was measured using a moisture detector (GAC2100AGRI, DICKEY-john, Minneapolis city, MN, United States). Spikelets per panicle, spikelets fertility and spikelets per m^2^ were measured. The panicles were hand-threshed and the filled grains were separated by submerging them in tap water. The filled grains were then oven-dried at 70°C to constant weight for determining grain weight. Culm diameter and culm wall thickness were measured with a digital caliper (accuracy of 0.01 mm) at the middle of the fourth internode from the top. Plant height was measured with a ruler from the base to the tip of the panicle excluding awns for at least 15 representative main stems. Gravity center height refers to the distance from the culm base to the balance fulcrum with panicle, leaf, and sheath.

### Measurement of Lodging-Related Index and Stem Physical Properties

Lodging-related traits and morphological parameters were determined at 20 days after heading. At least 10 main stems were collected and used for analysis. The lodging index and stem physical properties included the bending load, SL (the length from the broken point to the panicle top), FW (the fresh weight from the broken point to the panicle top). The bending load was measured at the fourth internodes from the top with AIKON (RX-5, Japan) at a distance of 6 cm according to a previous report (Ookawa, 1992). The lodging-related parameters were collected according to Ookawa’s description (Ookawa et al., 2010) as follows: (1) Bending moment by whole plant (g.cm) = SL (cm) × FW (g); (2) Breaking strength (g.cm) = 1/4 × breaking load (Kg) × L (Distance between fulcra (cm) × 10^3^); (3) Lodging index (%) = Bending moment by whole plant (g.cm)/Breaking strength (g.cm) × 100; (4) Cross-section modulus (mm^3^) = π/32 × (a_1_^3^b_1_ - a_2_^3^b_2_)/a_1_, where a_1_ is the outer diameter of the minor axis and a_2_ is the inner diameter of the minor axis; b_1_ is the outer diameter of the major axis and b_2_ is the inner diameter of the major axis; and (5) Bending stress (g mm^-2^) = Breaking strength/Cross section modulus.

### Scanning Electron Microscopy

The second basal internodes were collected at heading stage and fixed in 3% glutaraldehyde. The samples were prepared according to Mou’s description (Mou et al., 2000), with some modifications. Approximately 100 μm hand-cut transverse sections were critical point-dried, sputter-coated with gold, then imaged with a scanning electron microscope (S-3000N; Hitachi, Tokyo, Japan).

### Histochemical Staining of Transverse Sections

The fourth internodes from the top were collected at the heading stage and fixed in FAA solution (70% ethanol, 90 ml; acetic acid, 5 ml; and formaldehyde, 5 ml). To observe the lignified cell walls, phloroglucinol staining was performed according to standard protocols (Ookawa et al., 2014). First, the approximately 100 μm hand-cut sections were treated with 1% phloroglucinol solution (ethanol: water = 7:3 (V/V); Sigma) for 10 min, washed with 18% HCl for 5 min and then observed using fluorescence microscopy (Leica, Wetzlar, Germany) under a light microscope. For the cellulose accumulation observation, the approximately 100 μm transverse sections were treated with 0.05% calcofluor white (Sigma) for 2 min and then observed using a fluorescent microscope (Leica, Wetzlar, Germany).

### Measurement of Non-structural Carbohydrate, Sucrose, Lignin, and Cellulose Contents

The fourth internodes from the top were collected at 20, 10 days before heading; and 0, 20 days after heading, oven-dried at 105°C for 30 min and then oven-dried at 70°C to a constant weight, and then milled into powder to determine the non-structural carbohydrate, sucrose, lignin, and cellulose contents. The non-structural carbohydrate, sucrose, lignin, and cellulose content measurements were described by Updegraff (1969); Yoshida et al. (1976), Ishimaru et al. (2008), and Zhang et al. (2016a), respectively.

The non-structural carbohydrate content determination was performed using the following procedure: in brief, 0.1 g of each dried homogenized stem sample was extracted with distilled water for 2 h with boiling, and then they were extracted with perchloric acid (9.2 mol/L) three times. Anthrone colorimetry reagents were added to the extract and then a spectrophotometer was used to measure the absorbance at 620 nm.

The sucrose content determination was performed using the following procedure:

Briefly, 0.1 g of each dried homogenized stem samples were placed in test tubes with 15 ml, 80% ethanol was added, and then were water bathed at 80°C for 30 min, then centrifuged at 5000 r/min for 15 min, and the supernatant was transferred to a 50 ml volumetric flask. The above procedures were repeated three times. The volumetric flask constant volume is 50 ml. Finally, we added 0.1% resorcinol and 10 mol/L hydrochloride, and then were water bathed at 80°C for 30 min and then used the end product to measure the absorbance with a spectrophotometer at 480 nm.

A lignin content determination was performed using the following procedure; in brief, 0.5 g of dried homogenized stem samples were placed in test tubes with 10 ml, and 72% sulphuric acid was added slowly with stirring. The samples were then left at room temperature for 3 h with frequent stirring and transferred completely to a flask. Water was added to reach a dilution with a 3% sulphuric acid concentration, and then the samples were boiled for 4 h. The mixture was filtered through quantitative filter paper and washed with hot water until acid-free. The filter and lignin were dried at 105°C for 3 h until the weight was constant, then the weight was recorded. The lignin content was calculated using the following procedure: Lignin (%) = 100 W/S; where W is the weight of the insoluble lignin (g) and S is the weight of the dried stem sample (g). The cellulose content determination came before the following procedure. In brief, 0.1 g of dried homogenized stem samples were treated with 5 ml of acetic/nitric reagent in a tube, and then they were placed in a water bath boiling for 30 min, then centrifuged at 5000 r/min for 15 min, and the supernatant was then discarded. We repeated the above steps three more times until the residues were white. After the above treatments, 5 ml of 72% sulphuric acid was added to the tube and allowed to stand at room temperature for more than 12 h, then transferred to a 50 ml volumetric flask. The contents were washed several times with distilled water and transferred to a volumetric flask together to reach 50 ml. Finally, we added anthrone colorimetric reagents and then used the end product to measure the absorbance with a spectrophotometer at 620 nm.

### RNA Extraction and Real-Time Quantitative PCR (qRT-PCR) Analysis

The total RNA from the rice internode tissues was isolated using a Tiangen RNAprep Pure Plant kit (Beijing, China). After removing the genomic DNA contamination with DNase from the extracted RNA sample, the first-strand cDNA was synthesized by reverse transcriptase (Takara, Japan) according to the manufacturer’s protocol. qRT-PCR was performed on the Roche 480 Real-time PCR system (Switzerland). Quantitative PCR analysis was performed using the first-strand cDNA as templates with the SYBR Premix Ex TaqTM (Tli RNaseH Plus) (Takara, Japan), according to the cycling profile described in the instructions with the following cycling profile: denaturation, 95°C for 30 s, 1 cycle; PCR, 95°C for 5 s and 60°C for 30 s, 40 cycles; melting, 95°C for 5 s (4.4°C s/l cooling rate); and 60°C for 1 min (2.2°C s/l cooling rate), 95°C (0.11°C s/l cooling rate; continuous acquisition mode, 5 acquisitions per °C), 1 cycle. The 2^-ΔΔC_T_^ method was used to calculate the relative changes in expression levels (Livak and Schmittgen, 2001). The housekeeping β-actin gene was used as the internal reference. Each gene was tested using three replicates. The gene-specific primers are listed in **Table 1**.

**Table 1:** List of primer sets used for quantitative real-time quantitative PCR (RT-PCR).

Gene	Gene location	Forward primer 5′-3′	Reverse primer 5′-3′
*OsPAL*	Os02g0626100	ACCGCTTCGTGTATCTTCAG	AAGGATGGAATCGAGTAGCA
*OsCOMT*	Os08g0157500	GAAGGTGGTGGTGGTGGAGT	GCGTTGGCGTAGATGTAGGTG
*OsCCoAOMT*	Os08g0498100	CACCAACAAGACGCTGCTCAAG	CTCATCCGCCGACGACTGC
*OsCCR*	Os08g0277200	CGACCGCCACGACCTCTC	GCCCTGCCTCCGTCATCC
*OsCAD2*	Os02g0187800	CGACCAGAAGTTTGTGGTGAA	GAAGTGCTTCAGTGGGCTGTA
*OsCesA1*	Os05g0176100	TTGACTTGCACGATCGATACG	TCCCACATAAACTGGACCCTG
*OsCesA3*	Os07g0424400	GCATTTTTGCTACTGGCATCC	TCCCTGGAACAAAGCAAAGAG
*OsCesA4*	Os01g54620	GTTCGATGGCATTGATCGCA	CCACATAAACCGGACCTTGGA
*OsCesA7*	Os10g32980	CCGGATGGATGATTCTTGTTG	CCCCCAAAACACTTTTATCCC
*OsCesA8*	Os07g0208500	TGTTGAAGGTGCTGGATTCGA	TGAGAGTGGAGGCAACAAACG
*OsCesA9*	Os09g25490	AGGCCATCCATGTCATCAGCT	TTGAACCCCGTTAGGATGTCC
*OsActin*	Os03g0718100	CAATCGTGAGAAGATGACCC	GTCCATCAGGAAGCTCGTAGC

### Statistical Analysis

The figures were drawn with Origin 2015. The data are the means of three independent replicates. Statistically significant differences (^∗^ represents *P* < 0.05, ^∗∗^ represents *P* < 0.01) were determined by analyses of variance based on Student’s *t*-tests.

## Results

### Effects of the Shading Treatment on the Yield and Yield Components

Shading treatment had a significant effect on yield and yield components (**Table 2**). Under shading treatment, the number of panicles per m^2^ increased, while the spikelets per panicles and spikelets per m^2^ decreased in both cultivars. The panicles of Wuyunjing23 increased by 25.9 and 26.3% in 2015 and 2016, respectively; and that of the W3668 increased by 19.9% in 2015 and 10.5% in 2016. By contrast, the spikelets per panicles were significantly reduced in Wuyunjing23, by 14.0 % in 2015 and 36.2% in 2016, and that of the W3668 decreased by 31.7% in 2015 and 19.4% in 2016. A similar reduction trend occurred in both cultivars for both years. Additionally, shading results in grain weight and yield reduction. Compared with the control, the grain weight decreased 4.4 and 2.1% of Wuyunjing23 in 2015 and 2016, respectively; and that of W3668 by 6.9 and 4.6% in 2015 and 2016, respectively. A similar reduction trend was observed for the grain yield. However, the spikelets fertility had no significantly difference except Wuyunjing23 in 2015. The yield reduction under shading treatment was probably caused by the decreased spikelets per panicles, spikelets per m^2^ and grain weight.

**Table 2:** Effects of shading on grain yield and yield components of two japonica rice in 2015 and 2016.

Treatment	Panicles (m^2^)	Spikelets per panicles	Spikelets (10^3^/m^2^)	Spikelets fertility (%)	grain weight (mg)	Yield (t/ha)
**2015**						
**Wuyunjing23**						
Control	306.9	138.8**	42.6*	84.10	32.0*	11.5
Shading	386.4**	92.0	35.6	96.9*	30.6	10.6
W3668						
Control	393.3	104.2**	40.9	96.7	25.8**	10.2**
Shading	471.7**	71.1	33.6	95.2	24.0	7.7
**2016**						
**Wuyunjing23**						
Control	333.3	125.6**	41.9**	92.9	31.0**	12.0*
Shading	420.8**	80.1	33.7	96.1	30.3	9.8
W3668						
Control	478.3	88.3*	42.3	93.3	23.5**	9.3
Shading	528.6**	71.2	37.7	95.9	22.4	8.5

*Asterisks (^∗^) represent significant differences relative to the Control (^∗^*P* < 0.05; ^∗∗^*P* < 0.01).*

### Effects of the Shading Treatment on the Culm Diameter, Culm Wall Thickness, Gravity Center Height and Plant Height

The morphological traits were analyzed to evaluate the agronomic effects respond to shading. As shown in **Table 3**, shading treatment reduced culm diameter, culm wall thickness in both cultivars and years of the fourth internode from the top. While increased gravity center height and plant height in both cultivars over 2 years. These morphological characteristics showed similar trends under shading treatment.

**Table 3:** Effect of shading on culm diameter and culm wall thickness of the fourth internode from the top, gravity center height and plant height in two japonica rice.

Treatments	Clum diameter (mm)	Culm wall thickness (mm)	Gravity center height (cm)	Plant height (cm)
**2015**				
**Wuyunjing23**				
Control	4.36	0.90**	43.51	94.79
Shading	4.32	0.75	45.57**	100.60**
W3668				
Control	3.79**	0.70**	45.34	97.01
Shading	3.42	0.52	46.08	99.82**
**2016**				
**Wuyunjing23**				
Control	4.67**	0.86**	34.90	82.15
Shading	4.41	0.74	38.43**	85.50*
W3668				
Control	3.89*	0.69**	38.41	86.87
Shading	3.74	0.57	40.86**	89.87

*Asterisks (^∗^)represent significant differences relative to the Control under shading treatment (^∗^*P* < 0.05; ^∗∗^*P* < 0.01).*

### Effects of the Shading Treatment on the Breaking Strength, Bending Stress, and Bending Moment in the Whole Plant, and the Lodging Index

The culm mechanical strengths (which were determined as the breaking strength) were determined at the fourth internodes from the top of the rice plants, where lodging occurred most easily. Both Wuyunjing23 and W3668 experienced an increased lodging index under shading treatment in both years. The lodging index of Wuyunjing23 increased by 38.0% in 2015 and 4.5% in 2016, and the lodging index of W3668 increased by 22.9% in 2015 and 32.6% in 2016. By contrast, the breaking strength was significantly reduced in Wuyunjing23, by 26.7% in 2015 and 21.2% in 2016, and that of the W3668 decreased by 32.6% in 2015 and 27.4% in 2016. A similar trend in the shading effects on the bending stress was observed in both cultivars and both years. Shading treatment resulted in a significant reduction in the bending moment by the whole plant in W3668 in 2015 and Wuyunjing23 in 2016. Wuyunjing23 had higher breaking strength and bending stress, leading to a lower lodging index (**Table 4**).

**Table 4:** Effects of shading on breaking strength (M), bending stress (BS), bending moment by whole plant (WP) and lodging index (LI) of the fourth internode from the top in two japonica rice at 20 days after the heading stage.

Treatments	M (g cm)	BS (g cm)	WP (g cm)	LI (%)
**2015**				
**Wuyunjing23**				
Control	2780.7**	4171.5**	1741.2	62.8
Shading	2041.0	3411.8	1765.5	86.6**
W3668				
Control	1557.3**	3688.9	1298.7**	83.5
Shading	1049.0	3601.2	1077.0	102.7**
**2016**				
**Wuyunjing23**				
Control	2679.9**	3389.2**	1179.7**	44.0
Shading	2111.4	3328.1	972.0	46.1
W3668				
Control	1289.0**	2920.9	783.9	60.8
Shading	936.0	2558.3	775.1	82.8**

*Asterisks (^∗^) represent significant differences relative to the Control under shading treatment (^∗^*P* < 0.05; ^∗∗^*P* < 0.01).*

### Effect of the Shading Treatment on Microstructure Characteristics

**FIGURE 1:**
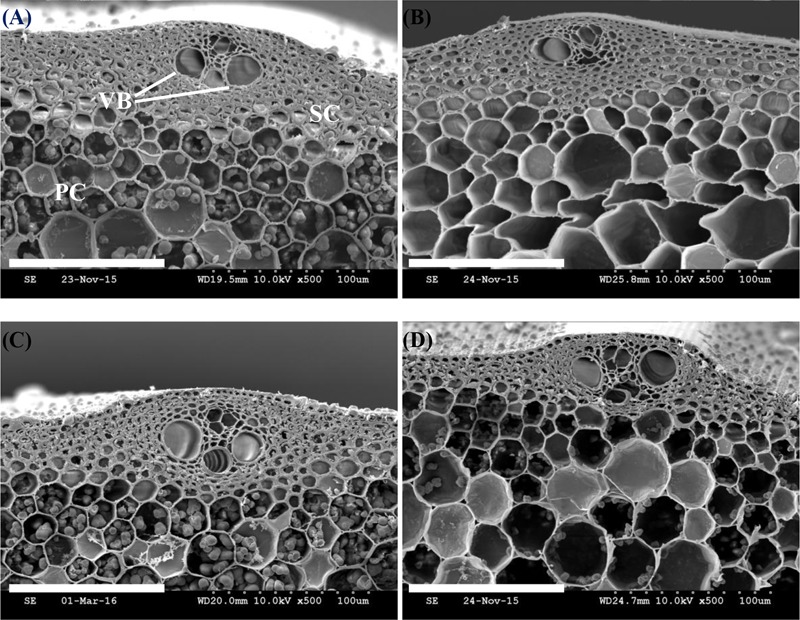
Effect of shading on scanning electron micrographs (SEM) in the microstructures of sclerenchyma cells and vascular bundle (VB) in the transverse section of the fourth internode from the top. PC, parenchyma cells; SC, sclerenchyma cells. **(A,B)** Cross-section of culm tissue in Wuyunjing23 under the Control and Shading treatment, respectively; **(C,D)** Cross-section of the culm tissue in W3668 under the Control and Shading treatment, respectively. Bar = 100 μm.

Electron microscope images of transverse sections were scanned to observe the stem development. Compared with W3668, the sclerenchyma cells layers of Wuyunjing23 were thicker (**Figure 1**). Shading altered the minute structures, such as the inner sclerenchyma cell layer, leading to the presence of more hollow cells in parenchyma cells tissues and thin sclerenchyma cell tissues for both cultivars (**Figures 1B,D**). The results showed that when shading from the jointing to the heading stages, it altered the stem cell wall microstructure characteristics and the component concentration.

### Effect of the Shading Treatment on Lignin and Cellulose Arrangement and Localization

**FIGURE 2:**
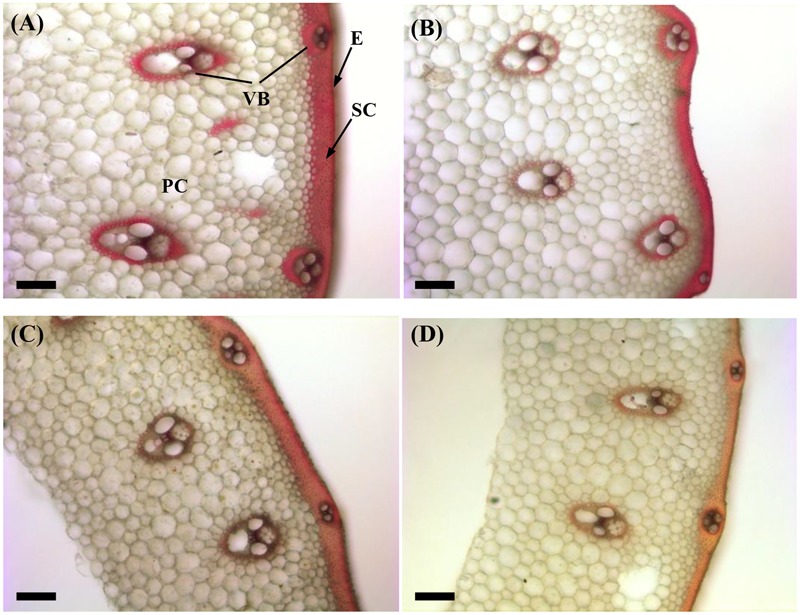
Effect of shading on Wiesner staining of the transverse internode section of the fourth internode from the top of Wuyunjing23 and W3668 plants. **(A,B)** Phloroglucinol staining of Wuyunjing23 under Control and Shading, respectively; **(C,D)** phloroglucinol staining of W3668 under Control and Shading, respectively. Bars = 100 μm. E, epidermis; PC, parenchyma cells; SC, sclerenchyma cells; VB, vascular bundle.

The structural carbohydrates lignin and cellulose contributed to the culm mechanical strength. As shown in **Figure 2**, the red-pink phloroglucinol staining of shading treatment samples was lighter than that of the Control for both cultivars (**Figures 2B,D**). These findings indicated that shading decreased the amount of lignin deposition in the sclerenchyma cells and around the vascular bundle sheath, as led by relatively poorer lignifications and more poorly developed secondary cell walls.

**FIGURE 3:**
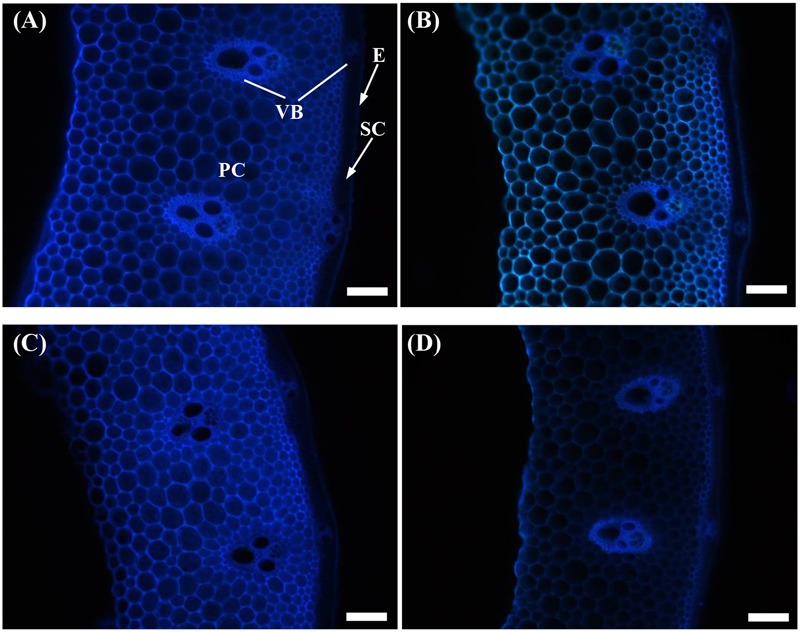
Effect of shading on calcofluor staining of the transverse internode section of the fourth internode from the top of Wuyunjing23 and W3668 plants. **(A,B)** Calcofluor staining of Wuyunjing23 under Control and Shading, respectively; **(C,D)** calcofluor staining of W3668 under Control and Shading, respectively. E, epidermis; PC, parenchyma cells; SC, sclerenchyma cells; VB, vascular bundle. Bars = 100 μm.

Similarly, the cellulose fluorescence intensity in the parenchyma cells of the shading treatment samples was weaker than that of the Control for both cultivars (**Figures 3B,D**). This finding was indicated by the lower densities of cellulose distribution under shading treatment.

### Effects of Shading Treatment on the Non-structural Carbohydrate, Sucrose, Lignin, and Cellulose Contents

**FIGURE 4:**
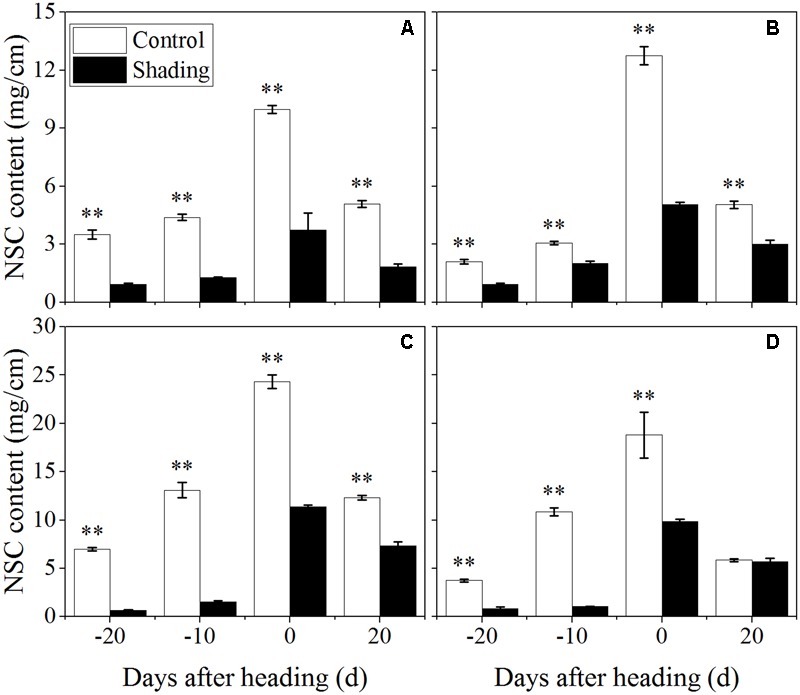
Effect of shading on NSC (non-structural carbohydrate) content in the culm tissue of the fourth internode from the top from 20 days before heading to 20 days after heading in Wuyunjing23 **(A,C)** and W3668 **(B,D)**. (**A,B**: 2015; **C,D**: 2016) Asterisks (^∗^) represent significant differences relative to the Control under different stages of culm growth (^∗^*P* < 0.05; ^∗∗^*P* < 0.01).

Furthermore, the contents of non-structural carbohydrates, sucrose, lignin, and cellulose in the fourth internodes from the top were investigated to distinguish among the changes in the cell wall component concentrations under shading in a quantitative manner. Shading was shown to decrease the non-structural carbohydrate content in both Wuyunjing23 and W3668 in both years (**Figure 4**). There were decreases of 73.3, 70.9, 62.5, and 63.8%; and 90.8, 88.1, 53.2, and 40.7% for Wuyunjing23 in 2015 and 2016 (**Figures 4A,C**), respectively; a similar reduction was observed in non-structural carbohydrate contents, with differences of 55.5, 34.0, 60.4, and 40.6%; and 78.3, 90.4, 47.5, and 3.3% for W3668 in 2015 and 2016 (**Figures 4B,D**).

**FIGURE 5:**
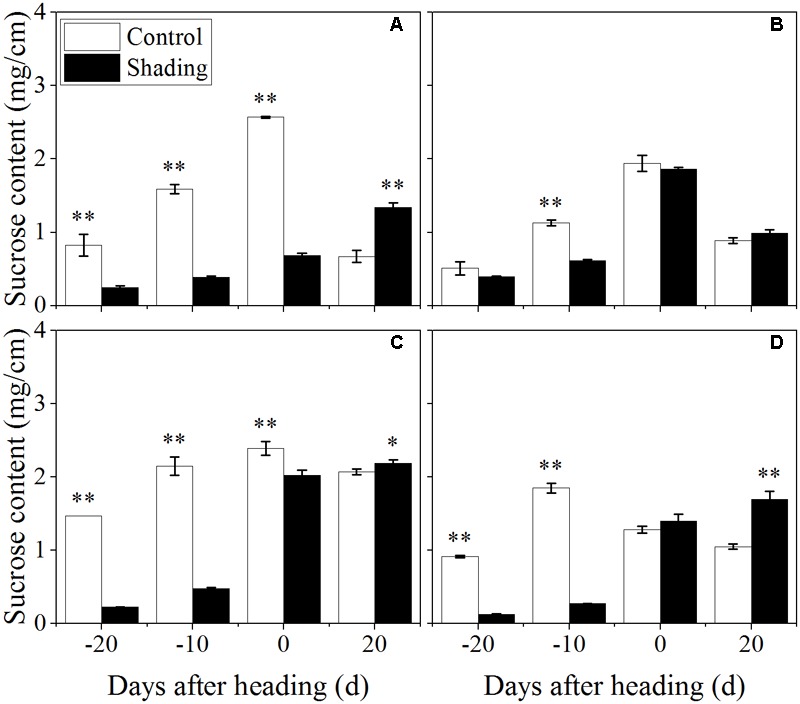
Effect of shading on sucrose content in the culm tissue of the fourth internode from the top from 20 days before heading to 20 days after heading in Wuyunjing23 **(A,C)** and W3668 **(B,D)**. (**A,B**: 2015; **C,D**: 2016) Asterisks (^∗^) represent significant differences relative to the Control under different stages of culm growth (^∗^*P* < 0.05; ^∗∗^*P* < 0.01).

The sucrose content was observed reduced and then increased from 20 days before heading to 20 days after heading under shading treatment (**Figure 5**). As compared to control, the sucrose content decreased at 20, 10 days before heading, and was observed an increasing trend at 20 days after heading. These data suggesting that shading decreased the sucrose content at elongation stage, while increased sucrose content at 20 days after heading.

**FIGURE 6:**
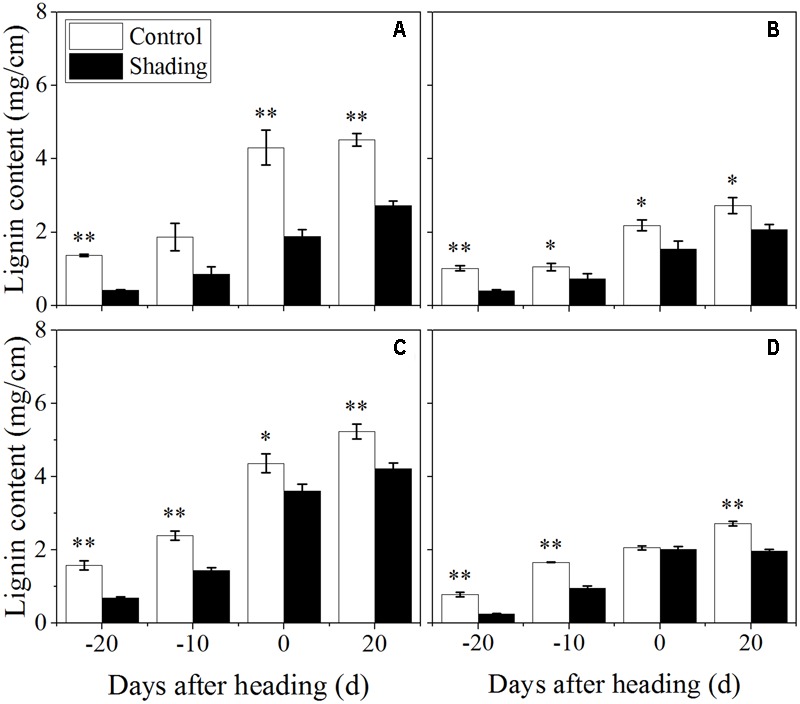
Effect of shading on lignin content in the culm tissue of the fourth internode from the top from 20 days before heading to 20 days after heading in Wuyunjing23 **(A,C)** and W3668 **(B,D)**. (**A,B**: 2015; **C,D**: 2016) Asterisks (^∗^) represent significant differences relative to the Control under different stages of culm growth (^∗^*P* < 0.05; ^∗∗^*P* < 0.01).

**FIGURE 7:**
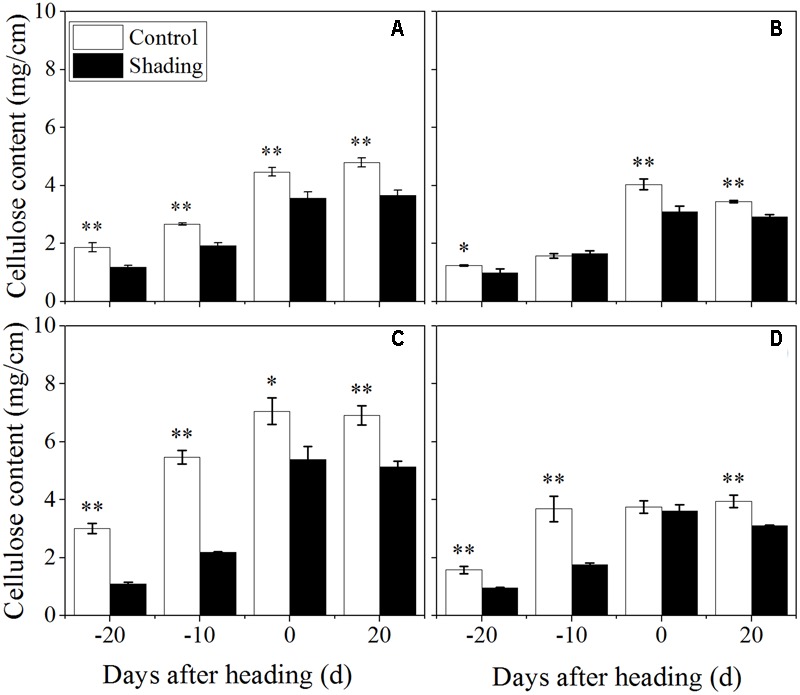
Effect of shading on cellulose content in the culm tissue of the fourth internode from the top from 20 days before heading to 20 days after heading in Wuyunjing23 **(A,C)** and W3668 **(B,D)**.(**A,B**: 2015; **C,D**:2016) Asterisks (^∗^) represent significant differences relative to the Control under different stages of culm growth (^∗^*P* < 0.05; ^∗∗^*P* < 0.01).

The shading treatment also decreased the lignin content in both Wuyunjing23 and W3668 in both years (**Figure 6**). There were decreases of 69.7, 54.1, 56.4, and 39.8%; and 56.7, 39.3, 17.1, and 19.4% for Wuyunjing23 in 2015 and 2016 (**Figures 6A,C**), respectively; a similar reduction was observed in the lignin content with changes of 60.2, 30.3, 29.4, and 24.4 %; and 66.8, 42.1, 2.4, and 27.7% for W3668 in 2015 and 2016 (**Figures 6B,D**). The cellulose content under shading was decreased significantly in Wuyunjing23, and it decreased by 36.6, 27.8, 20.3, and 23.4%; and 63.6, 59.9, 23.5, and 25.6% in 2015 and 2016, respectively (**Figures 7A,C**). Similarly, the cellulose content of W3668 was decreased under shading treatment, by 20.5, 4.5, 23.3, and 15.2%; and 39.2, 52.3, 3.8, and 21.1%, respectively (**Figures 7B,D**).

The non-structural carbohydrate, lignin, and cellulose concentrations responded to shading treatment in a consistent fashion in the two cultivars. These concentrations were consistent with the lignin and cellulose staining (**Figures 2, 3**) and were in accordance with the microstructure characteristics (**Figure 1**). However, the sucrose content was observed a reduction and then increment trend. These data suggesting that the sucrose content was strongly regulated by shading treatment. These results also indicated that shading decreased the lignin and cellulose content and thus affected the culm mechanical strength.

### Effect of Shading Treatment on Lignin and Cellulose Biosynthesis Gene Expression

**FIGURE 8:**
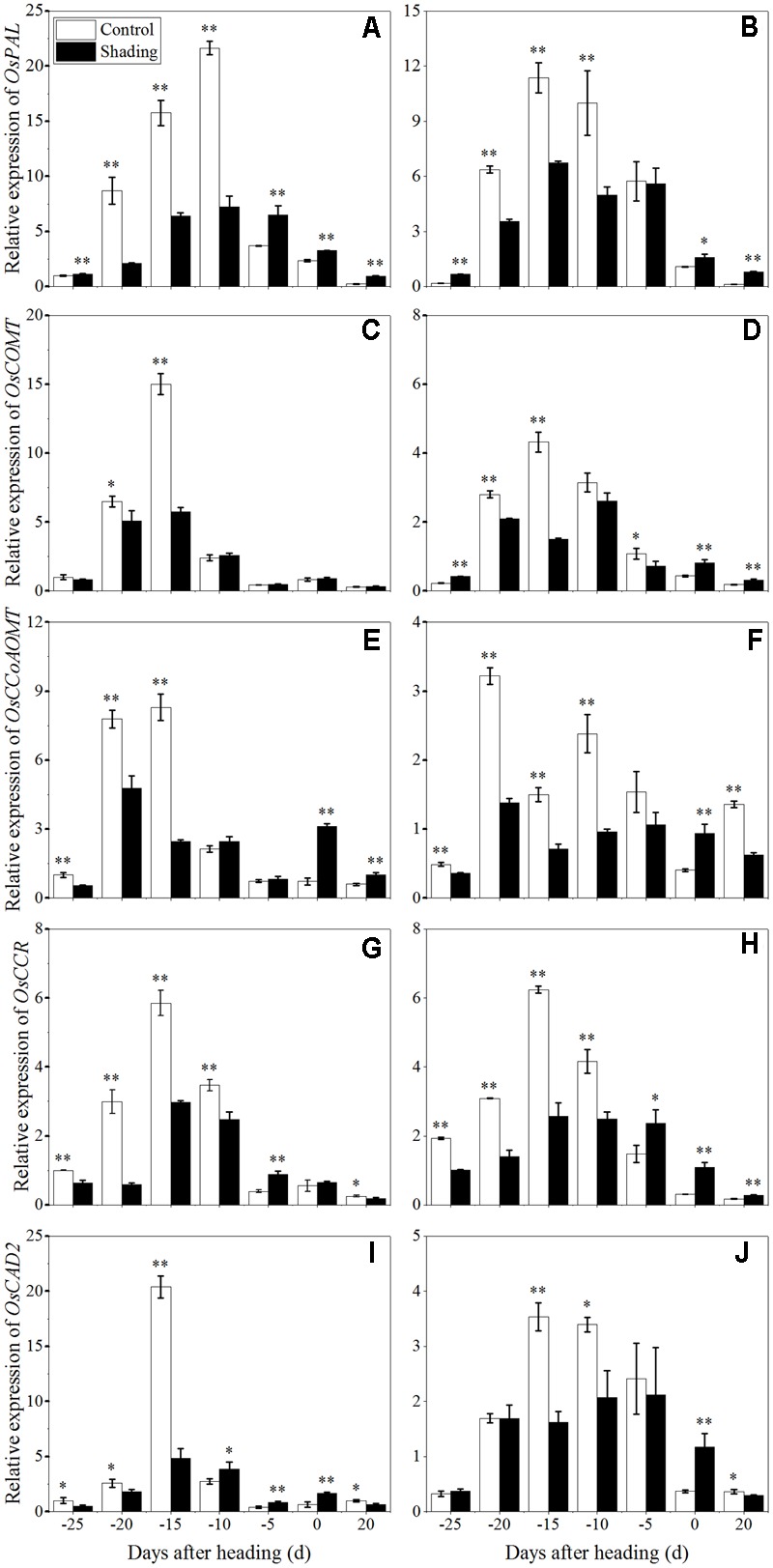
Effects of shading on the expression of *OsPAL* in Wuyunjing23 **(A)** and W3668 **(B)**, the expression of *OsCOMT* in Wuyunjing23 **(C)** and W3668 **(D)**, the expression of *OsCCoAOMT* in Wuyunjing23 **(E)** and W3668 **(F)**, the expression of *OsCCR* in Wuyunjing23 **(G)** and W3668 **(H)** and the expression of *OsCAD2* in Wuyunjing23 **(I)** and W3668 **(J)** in from 25 days before heading to 20 days after heading. Total RNA was isolated from culm tissues of the fourth internode from the top of the plant. β-actin was used as a loading control, and the value obtained from the Control treatment at 25 days before heading was arbitrarily set as 1.0. Real-time PCR was performed in triplicate, and the mean values with SD are shown. Asterisks represent significant differences relative to the Control treatment under different stages of culm growth (^∗^*P* < 0.05; ^∗∗^*P* < 0.01).

The expression of the genes known to be involved in lignin and cellulose synthesis in culms was investigated with qRT-PCR to characterize the role of shading in the process. The expression levels of *OsPAL* (*phenylalanine ammonia-lyase*), *OsCOMT* (*Caffeic acid 3-O-methyltransferase*), *OsCCoAOMT* (*Caffeoyl coenzyme A 3-O-methyltransferase), OsCCR* (*Cinnamoyl-CoA reductase*), *OsCAD2* (*cinnamyl alcohol dehydrogenase*), which are involved in the phenylpropanoid pathway for lignin biosynthesis, were analyzed (**Figure 8**). *OsPAL* was found to have the highest expression levels at 10 and 15 days before heading in Wuyunjing23 and W3668, respectively. *OsCOMT, OsCCoAOMT, OsCCR*, and *OsCAD2* displayed the highest expression levels at 15 days before heading in both Wuyunjing23 and W3668, while *OsCCoAOMT* was highest at 20 days before heading in W3668. Compared with the shading treatment, the expression levels of *OsPAL, OsCOMT, OsCCoAOMT, OsCCR*, and *OsCAD2* were down-regulated significantly at most sampling times before the heading stage. These results support the conclusion that shading largely reduces lignin biosynthesis in culm tissue before the heading stage.

**FIGURE 9:**
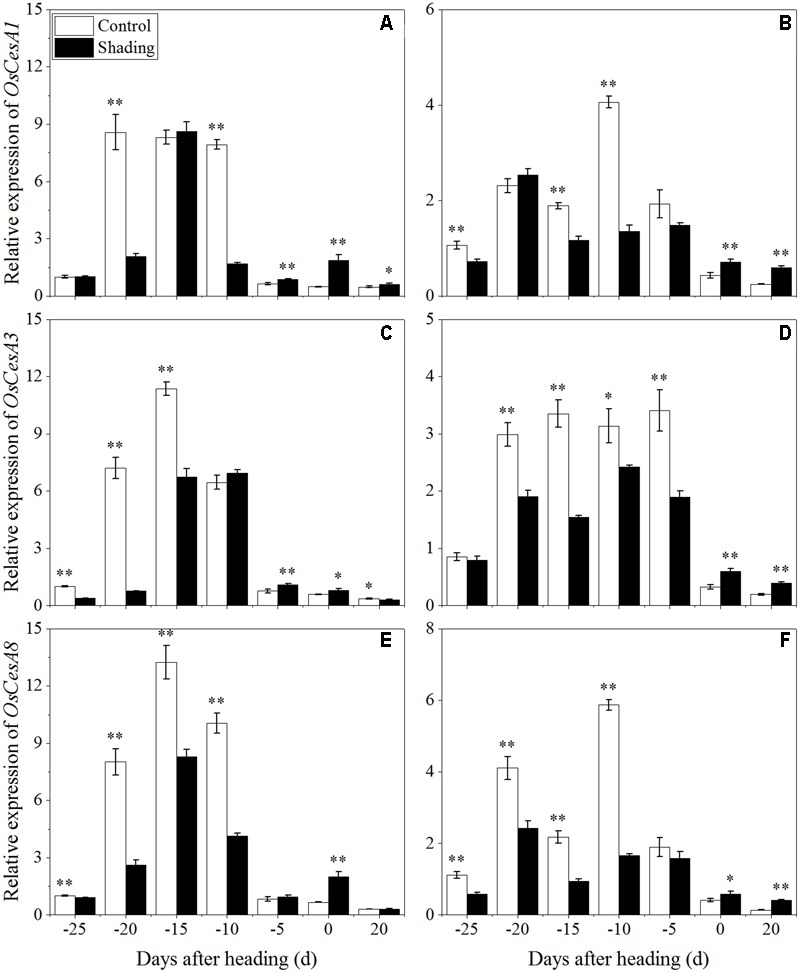
Effects of shading on the expression of *OsCesA1* in Wuyunjing23 **(A)** and W3668 **(B)**, the expression of *OsCesA3* in Wuyunjing23 **(C)** and W3668 **(D)**, and the expression of *OsCesA8* in Wuyunjing23 **(E)** and W3668 **(F)** from 25 days before heading to 20 days after heading. Total RNA was isolated from culm tissues of the fourth internode from the top of the plant. β-actin was used as a loading control, and the value obtained from the Control treatment at 25 days before heading was arbitrarily set as 1.0. Real-time PCR was performed in triplicate, and the mean values with SD are shown. Asterisks represent significant differences relative to the Control treatment under different stages of culm growth (^∗^*P* < 0.05; ^∗∗^*P* < 0.01).

**FIGURE 10:**
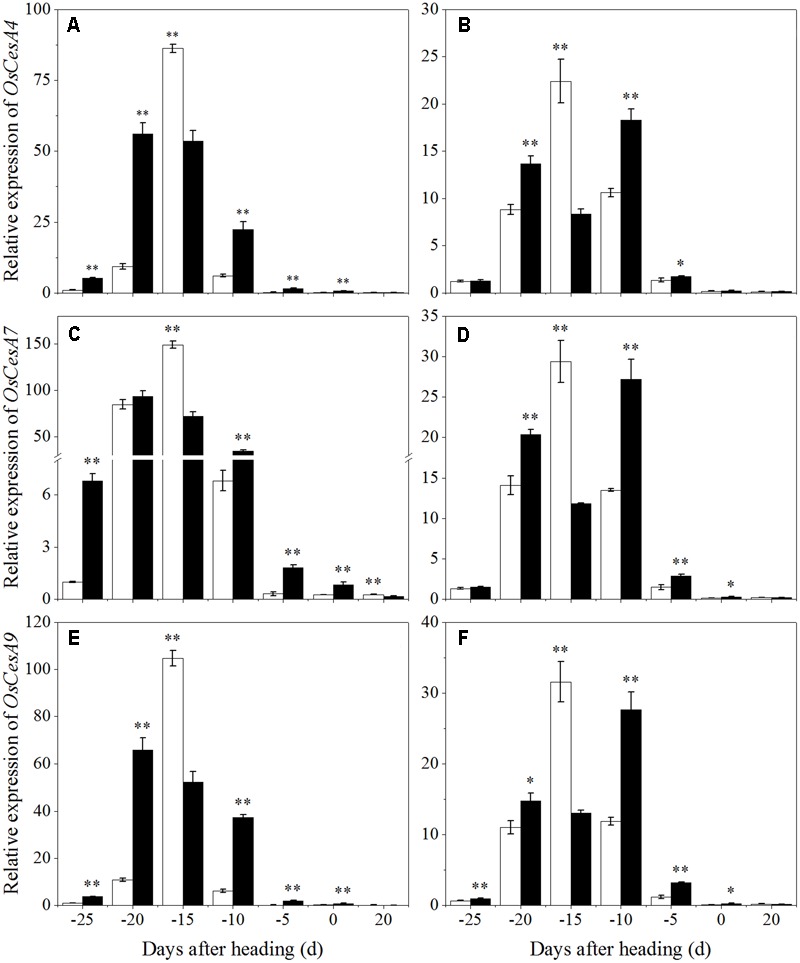
Effects of shading on the expression of *OsCesA4* in Wuyunjing23 **(A)** and W3668 **(B)**, the expression of *OsCesA7* in Wuyunjing23 **(C)** and W3668 **(D)**, and the expression of *OsCesA9* in Wuyunjing23 **(E)** and W3668 **(F)** from 25 days before heading to 20 days after heading. Total RNA was isolated from culm tissues of the fourth internode from the top of the plant. β-actin was used as a loading control, and the value obtained from the Control treatment at 25 days before heading was arbitrarily set as 1.0. Real-time PCR was performed in triplicate, and the mean values with SD are shown. Asterisks represent significant differences relative to the Control treatment under different stages of culm growth (^∗^*P* < 0.05; ^∗∗^*P* < 0.01).

To clarify the effects of shading on cellulose biosynthesis in cell walls, the expression levels of *OsCesA1* (*Cellulose synthases*), *OsCesA3* and *OsCesA8* (Wang et al., 2010) were studied, and they were primarily expressed in the primary cell wall. *OsCesA4, OsCesA7*, and *OsCesA9* (Kotake et al., 2011) were primarily expressed in secondary cell walls. As shown in **Figure 9**, *OsCesA1, OsCesA3*, and *OsCesA8* were up-regulated first and then down-regulated in both cultivars under shading treatment. Compared to the control, *OsCesA1* showed a lower expression level at 20 and 10 days before heading for Wuyunjing23, and at 25, 15, and 10 days before heading for W3668 under shading treatment, respectively. *OsCesA3* had a lower expression level at 25, 20, and 15 days; and at 25, 15, 10, and 5 days before heading in Wuyunjing23 and W3668 compared to the control, respectively. *OsCesA8* displayed a lower expression level at 25, 20, 15, and 10 days; and at 25, 20, 15, and 10 days before heading in Wuyunjing23 and W3668 compared to the control, respectively. Therefore, the cellulose biosynthetic pathway-related genes in the primary cell wall were strongly down-regulated before the heading stage. As shown in **Figure 10**, except at 15 days before heading, *OsCesA4, OsCesA7*, and *OsCesA9* were down-regulated, while at the other stage, *OsCesA4, OsCesA7*, and *OsCesA9* were up-regulated.

## Discussion

Solar radiation is an important environmental factor in plant growth and development. Shading leading to lower PAR was recognized as a plant stress. Several investigations have reported that shading on plant growth and yield in several crops (Beed et al., 2007; Liu and Tollenaar, 2009; Mo et al., 2015). Here we have found that shading had a significant effect on grain yield and yield components. Shading resulted in reduction of spikelets per panicles, spikelets per square meters, grain weight, and then resulted in grain yield reduction in the two cultivars over 2 years, which consist with previous studies in rice (Mo et al., 2015). These results demonstrate that yield and yield components were affected by shading treatment.

Previous studies have shown that shading leads to slender stems in rice (Liu et al., 2016), soybeans (Liu W. et al., 2015), and Arabidopsis (Yu et al., 2015) and increased lodging rates in maize (Xue et al., 2016) and wheat (Sparkes and King, 2008). Shading treatments decreased the dry weight per unit length and the rind penetration strength, suggesting that shading led to lower stem strength and increased lodging rates (Xue et al., 2016). In this study, shading during elongation up to the heading stage significantly reduced the stem-breaking strength and resulted in an increased lodging index. This result was related closely to the results of previous investigations in which shading decreased the stem mechanical strength and increased the lodging rate.

Plant height and gravity center height were important morphologic traits related to lodging (Chandler, 1969; Kashiwagi et al., 2008). In the present study, shading increased plant height, gravity center height of japonica rice stems thereby increasing the risk of lodging. In addition, culm diameter and culm wall thickness were also primary factors related to lodging (Zuber et al., 1999; Islam et al., 2007). In this study, shading decreased culm diameter and culm wall thickness of the basal second internode under shading treatment, which was consistent of previous reports (Liu et al., 2016). Thus, the basal second internode of shading treated stems were more slender leading to lower mechanical strength, which increased the lodging index (**Table 4**).

The thicknesses of mechanical tissue layers and vascular bundle sheath cells are the primary sources of mechanical strength, and they are important for crop lodging resistance at the anatomical level (Zhang et al., 2016b). In cereal plants, sclerenchyma cell tissues and vascular bundle tissues provide mechanical support for plants (Li et al., 2009; Kong et al., 2013). In the present study, shading decreased the layers of sclerenchyma cells for the mechanical tissues and vascular bundle tissues (**Figure 1**), and the variation trends of the two varieties were consistent. Furthermore, shading also led to many hollow tissues in parenchymal cell tissues. The results showed that shading decreased the thickness of mechanical tissues and resulted in decreased mechanical strength.

The stem mechanical strength is primarily dependent on the cell wall components (Kashiwagi et al., 2008) lignin and cellulose, which are the primary biochemical components that are closely associated with stem mechanical strength and that are positively associated with lodging resistance (Li et al., 2013; Peng et al., 2014; Zhang et al., 2014). In this study, we noted that shading decreased the non-structural carbohydrates (**Figure 4**), lignin (**Figure 6**), cellulose accumulation (**Figure 7**), and sucrose content before heading (**Figure 5**). Combined with histochemical staining, the decreased lignin accumulation is primarily reflected in the sclerenchyma tissue cells (**Figure 2**). The decreased cellulose accumulation is primarily reflected in the parenchyma tissues (**Figure 3**), and the non-structural carbohydrates that filled in the parenchyma tissues were decreased. Lignin is known as the material that thickens secondary cell walls, and it provides mechanical support for plants; cellulose acts as a net support for plants, and non-structural carbohydrates fill the parenchyma tissues to provide stem plumpness. In addition, sucrose as the main photosynthetic products transported to other non-photosynthetic tissues (Rolland et al., 2001), sucrose not only provide substrate for cellulose synthesis (Amor et al., 1995), but also provide carbon source for lignin biosynthesis (Rogers et al., 2005). In the present study, shading declined sucrose accumulation before heading thereby. However, the sucrose concentration was higher at 20 days after heading; therefore, a lot of sucrose could not be used in cellulose or lignin biosynthesis, leading to the decrease of cellulose and lignin concentration. Thereby reduced carbon reduced carbon source for cellulose and lignin biosynthesis. Thus, the results showed that shading decreased the stem plumpness and stem mechanical strength, and thus it increased the lodging index.

Moreover, lignin or cellulose biosynthesis gene mutations caused lignin or cellulose accumulation and decreased mechanical strength. *OsCesA4, OsCesA7*, and *OsCesA9* encode secondary cell wall cellulose biosynthesis (Kotake et al., 2011), and its related mutation decreased the cellulose concentration and mechanical strength. *OsCesA1, OsCesA3*, and *OsCesA8* encode the components of primary cell wall cellulose biosynthesis (Tanaka et al., 2003; Wang et al., 2010). To gain insights into the physiological and molecular basis for the regulation of cellulose and lignin biosynthesis in rice tissues under shading, we detected the spatiotemporal expression pattern of selected cellulose and lignin biosynthesis genes. Previous studies showed that mutations in the rice cellulose biosynthesis genes resulted in loose cell walls and decreased mechanical strength (Tanaka et al., 2003). In addition, the rice brittle culms *BC1, BC3, BC5, BC10, BC12, BC14*, and *BC15* showed lower cellulose and lower mechanical strength (Aohara et al., 2009; Zhou et al., 2009; Hirano et al., 2010; Zhang et al., 2010, 2011; Wu et al., 2012). Cellulose is synthesized with sucrose as the substrate (Amor et al., 1995), while the sucrose metabolism was sensitive to shading. Shading decreased the sucrose and cellulose contents (Chen et al., 2014), suggesting that shading led to lower cellulose accumulation in cotton. However, these investigations did not provide explanations of the mechanisms underlying those results in rice for shading between structural carbohydrates and lodging resistance. *OsCesA1, OsCesA3*, and *OsCesA8*; and *OsCesA4, OsCesA7*, and *OsCesA9* genes are the major genes that are responsible for cellulose biosynthesis in the primary cell walls (Tanaka et al., 2003; Wang et al., 2010; Li et al., 2014) and secondary cell walls (Tanaka et al., 2003; Wang et al., 2010; Kotake et al., 2011; Li et al., 2014) of rice stems, respectively. Primary cell walls experience inward thickening formed by secondary cell walls. Cellulose accumulation within plants may mostly be regulated by cellulose biosynthesis genes. In the present research, the initial down-regulation of *OsCesA1, OsCesA3*, and *OsCesA8* was probably caused by shading, and the next up-regulation of cellulose biosynthesis genes was a self-regulating phenomenon. The reduced cellulose content (**Figure 7**) was consistent with the less strong calcofluor staining for cellulose (**Figure 3**). Similar findings have been reported in cotton (Chen et al., 2014). Herein, we also observed the compensatory regulation of cellulose biosynthesis genes. Shading decreased the transcript levels of primary cell walls while increasing the transcript levels of genes encoding cellulose in secondary cell walls, but it decreased the concentration of cellulose. In fact, the compensatory regulation of cellulose biosynthesis is firmly established as a mechanism for adapting to adverse environment conditions such as shading. The reduction of cellulose accumulation under shading in parenchyma cells is primarily due to the down-regulation of *OsCesA1, OsCesA3*, and *OsCesA8* expression (**Figure 9**) before heading, and it resulted in reductions in mechanical strength to some extent. This research also found that cellulose accumulation in mechanical tissues and the periphery of vascular bundles exhibited little variation under shading compared to the control, which was primarily due to the up-regulation of *OsCesA4, OsCesA7*, and *OsCesA9* expression (**Figure 10**) before heading.

Moreover, Lignin can enhance stem mechanical strength by incorporating into cell walls. As previous reported, the expression of *PAL, COMT, CCoAOMT*, CCR and *CAD* and genes, which are involved in lignin biosynthesis, was associated with culm rigidity and lodging resistance (Ma, 2009, 2010; Ma and Luo, 2015). The functions of lignin in maintaining stem mechanical strength and lodging resistance were further reported in buckwheat (Wang C. et al., 2015), rice (Li et al., 2009; Zhang et al., 2014), and sorghum (Sattler et al., 2009). In addition, lignin is a classic phenolic compounds, and it was sensitive to light (Koyama et al., 2012). In tea, shading influenced the phenylpropanoid biosynthesis pathway, implying that light was an important environment factor that affected phenylpropanoid metabolism, and lignin may be the primary phenylpropanoid metabolic product of light induction (Wang et al., 2012a,b). This finding implied that lignin biosynthesis is affected by light environments (Koyama et al., 2012). However, there is no direct proof to demonstrate that light influenced lignin accumulation in rice stems. In the present study, we found that shading decreased the lignin content in sclerenchyma cells and the vascular bundles of the fourth internodes (**Figure 2**). The down-regulation of *OsPAL, OsCOMT, OsCCoAOMT, OsCCR*, and *OsCAD2* genes (**Figure 8**) was observed at 20–10 days before heading (the early or mid-period of jointing stage) under shading, while this stage was vital stage of lignin accumulation and cell wall thickening. These findings implied that shading restrained the lignin biosynthesis gene expression at this stage, which may be a lignin biosynthesis response to shading, and the restraining trends in gene expression under shading were consistent with the decreased lignin content (**Figure 6**) and the phloroglucinol staining of lignin (**Figure 2**). However, the later period, lignin biosynthesis gene expression was observed to be up-regulated under shading, it may be a self-regulating phenomenon or a shade-avoidance syndrome to compensate for the shading condition. These findings indicated that shading down-regulated the lignin biosynthesis genes in the early jointing stages, resulting in lignin accumulation deficits in mechanical tissues and vascular bundles, leading to an increased lodging index and decreased lodging resistance.

In summary, shading treatment decreased sucrose concentration and resulted in decreased carbon supply for cellulose and lignin biosynthesis, thus affected the expression of the key genes involved in cellulose and lignin biosynthesis. The alteration of gene expression restrain caused a decrease in the cellulose and lignin contents. Therefore, we believe that the above mentioned results are valuable for providing underlying mechanism of the decreased stem mechanical strength under shading condition.

## Author Contributions

Conceived and designed the experiments: LW, GL, and YD. Performed the experiments: LW, WZ, and JZ. Collected samples: LW, WZ, JZ, FW, and EC Analyzed the data: LW, WZ, JZ, and GL Provided good advice: ZL, ST, CD, LC, and SW. Drafted the manuscript: LW. Revised the manuscript and gave final approval of the version to be published: LW and GL.

## Conflict of Interest Statement

The authors declare that the research was conducted in the absence of any commercial or financial relationships that could be construed as a potential conflict of interest.
